# Supplementary figures and images for: Expression, localization, and function of P4HB in the spermatogenesis of Chinese mitten crab (Eriocheir sinensis)
Source: PeerJ. 2023 Jun 14;11:e15547. doi: 10.7717/peerj.15547 (PMC10276555; doi:10.7717/peerj.15547)

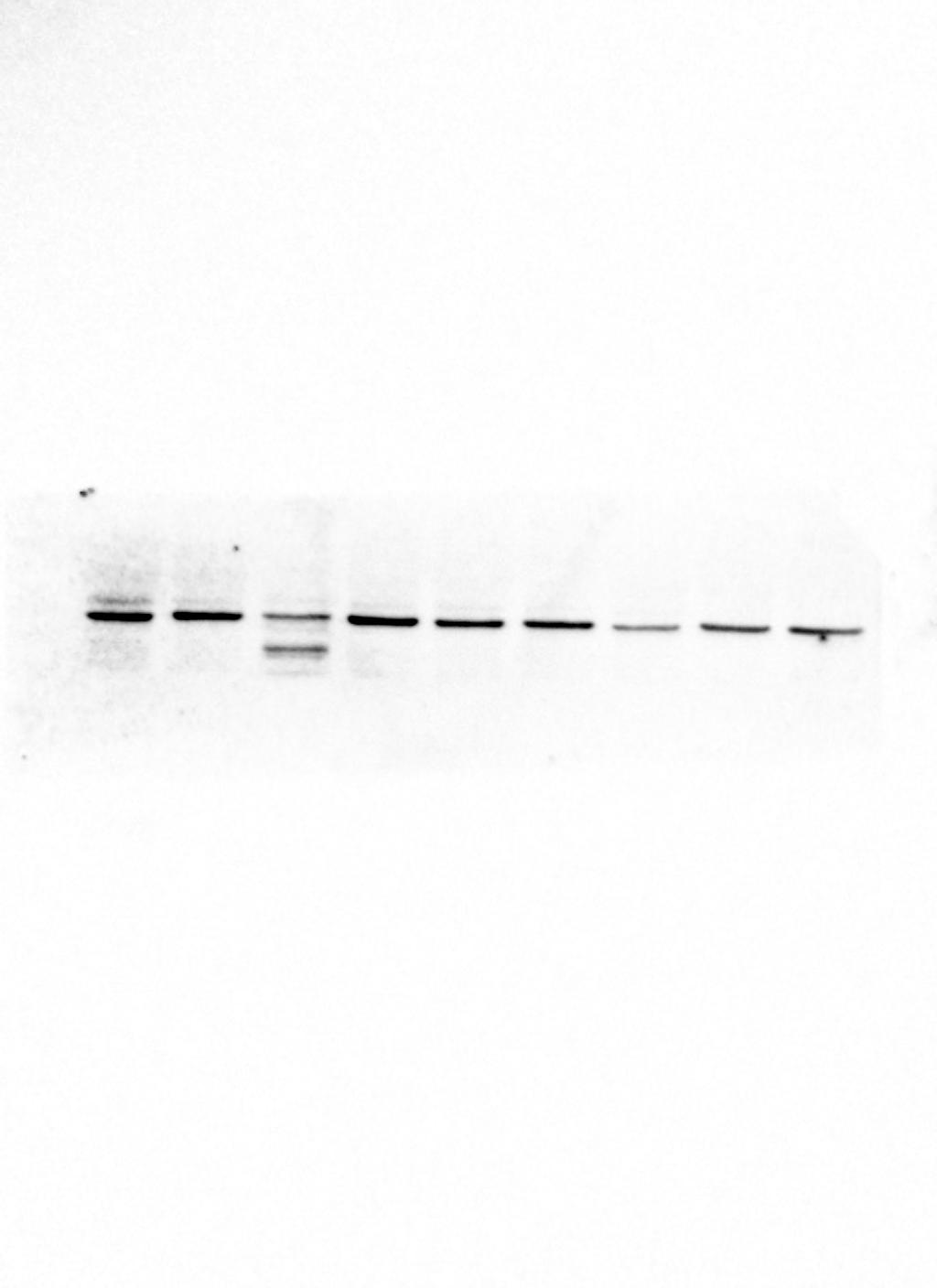

Supplement: Supplemental Information 3 [file peerj-11-15547-s003.jpg]

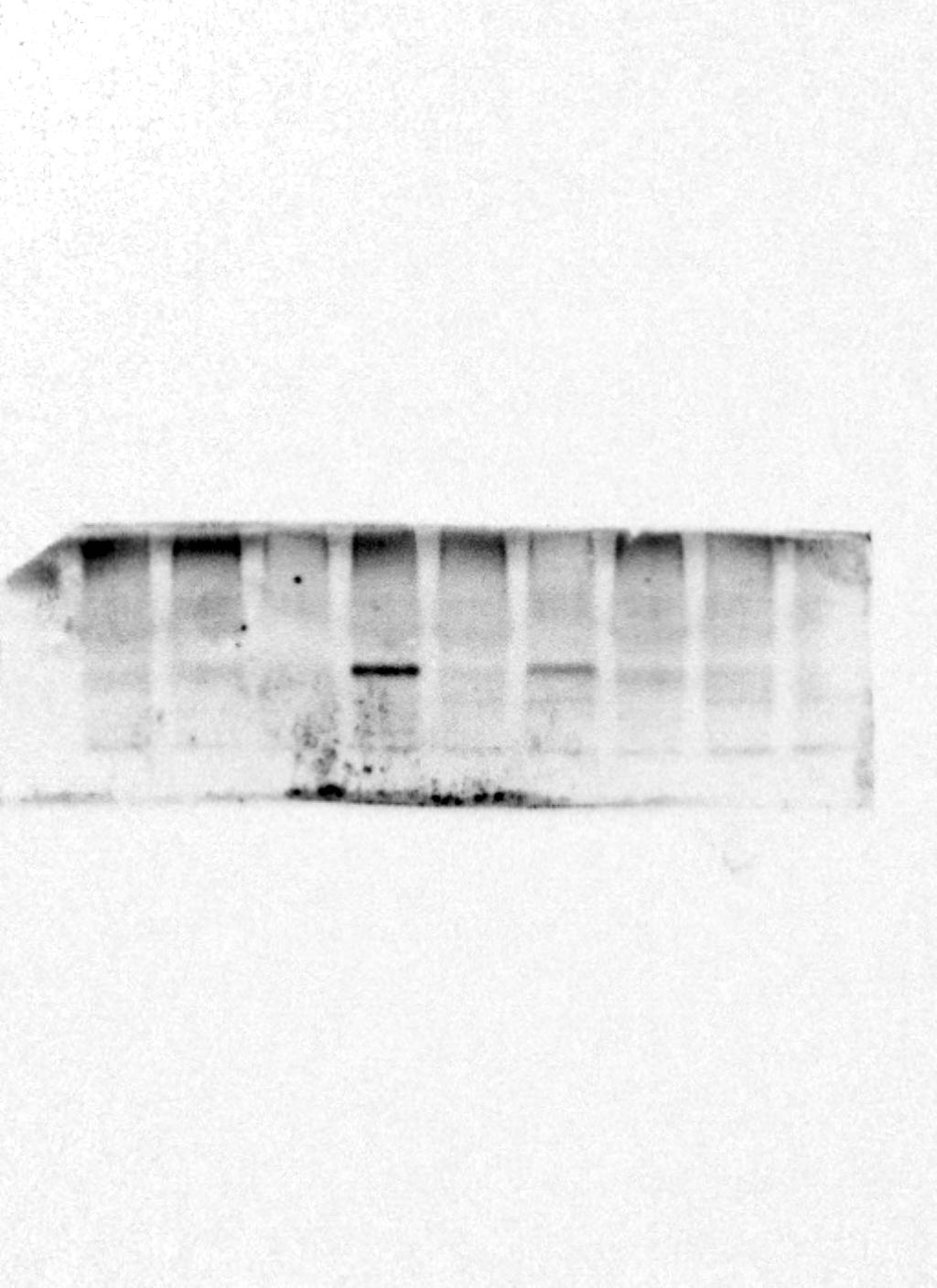

Supplement: Supplemental Information 4 [file peerj-11-15547-s004.jpg]
